# Synthetic sex-aggregation pheromone of *Lutzomyia longipalpis*, the South American sand fly vector of *Leishmania infantum*, attracts males and females over long-distance

**DOI:** 10.1371/journal.pntd.0008798

**Published:** 2020-10-20

**Authors:** Mikel A. González, Melissa Bell, Cristian F. Souza, Rafael Maciel-de-Freitas, Reginaldo P. Brazil, Orin Courtenay, James G. C. Hamilton

**Affiliations:** 1 Division of Biomedical and Life Sciences, Faculty of Health and Medicine, Lancaster University, Bailrigg, Lancashire, United Kingdom; 2 Laboratório Doenças Parasitárias, Instituto Oswaldo Cruz—Fiocruz, Pavilhão Arthur Neiva, Av. Brasil, Rio de Janeiro, RJ, Brazil; 3 Laboratório de Mosquitos Transmissores de Hematozoários, Instituto Oswaldo Cruz, Pavilhão Carlos Chagas, Av. Brasil, Rio de Janeiro, RJ, Brazil; 4 Zeeman Institute and School of Life Sciences, University of Warwick, Coventry, United Kingdom; National Institutes of Health, UNITED STATES

## Abstract

**Background:**

In South America the sand fly *Lutzomyia longipalpis* is the predominant vector of *Leishmania infantum*, the parasite that causes canine and human visceral leishmaniasis. Co-location of synthetic male sex-aggregation pheromone with an insecticide provided protection against canine seroconversion, parasite infection, reduced tissue parasite loads, and female sand fly densities at households. Optimising the sex-aggregation pheromone + insecticide intervention requires information on the distance over which female and male *Lu*. *longipalpis* would be attracted to the synthetic pheromone in the field.

**Methodology/Principal findings:**

Wild *Lu*. *longipalpis* were collected at two peridomestic study sites in Governador Valadares (Minas Gerais, Brazil). Sand flies were marked with coloured fluorescent powder using an improved protocol and then released into an existing domestic chicken shed at two independent sites, followed by recapture at synthetic-pheromone host-odour baited traps placed up to 30 metres distant from the release point.

In total 1704 wild-caught *Lu*. *longipalpis* were released into the two chicken sheds. Overall 4.3% of the marked flies were recaptured in the pheromone baited experimental chicken sheds compared to no marked flies recaptured in the control sheds. At the first site, 14 specimens (10.4% of the marked and released specimens) were recaptured at 10m, 36 (14.8%) at 20m, and 15 (3.4%) at 30m. At the second site, lower recapture rates were recorded; 8 marked specimens (1.3%) were recaptured at 5 and 10m and no marked specimens were recaptured at 15m. Approximately 7x more marked males than females were recaptured although males were only 2x as common as females in the released population. 52% of the marked *Lu*. *longipalpis* were collected during the first night of sampling, 32% on the second night, and 16% on the third night.

**Conclusions/Significance:**

The study established that both male and female sand flies can be attracted to the synthetic sex-aggregation pheromone in the presence of host odour over distances up to at least 30m in the field depending on local environmental and meterological conditions.

## Author summary

Visceral leishmaniasis (VL) is a disease caused by an insect transmitted protist parasite. In South America and Brazil in particular, it causes significant morbidity and mortality, with thousands of human cases and deaths reported every year. Domestic dogs are the most important source of human infection. Controlling the sand flies that transmit the parasite is one way to reduce the number of VL cases and recent research has shown the potential for a new pheromone-based approach to vector control. In a recent cluster-randomised control trial, co-location of a synthetic copy of the male sand fly sex-aggregation pheromone with pyrethroid insecticide reduced numbers of sand flies in households and provided protection for dogs against leishmaniasis infection incidence. The current study was carried out to determine the distance over which the synthetic sex-aggregation pheromone could attract *Lu*. *longipalpis* in a peridomestic environment in a Brazilian city. Male and female *Lu*. *longipalpis* were attracted up to 30m in one night towards a source of the pheromone. This information will help to inform the optimisation of placement of sex pheromone/insecticide intervention in Brazil.

## Introduction

Pheromones in Diptera are diverse and complex molecules and over the past two decades, elucidation of their composition and structure has concentrated predominantly on agricultural pest species [1]. In the Diptera order, the best studied pheromones are in the Cyclorrhapha taxon, e.g. Agromyzidae (leaf miner flies) and Tephritidae (fruit flies); in the latter, male produced long-range attractants have been studied in more than 30 species. Within nematoceran families, e.g. Cecidomyiidae (gall midges) and the Sciaridae (fungus gnats), female-produced contact (or short-range) attractants are widespread [1,2]. With respect to human and animal disease vectors, within the Psychodidae, subfamily Phlebotominae (sand flies), male-produced sex-aggregation pheromones of certain species attract both female and male conspecifics [3].

In the New World, Phlebotomine sand fly vector species are restricted to the genus *Lutzomyia* [4]. Throughout Latin America, the Phlebotomine sand fly *Lutzomyia longipalpis* s.l. is the main vector of *Leishmania infantum* (Kinetoplastida: Trypanosomatidae), a Protist parasite that causes visceral leishmaniasis (VL) in humans and canids [5]. This disease is a significant cause of morbidity and mortality in both humans and dogs in Brazil [6] and despite the substantial efforts made by the Brazilian Ministry of Health and State Authorities, the burden of VL in Brazil more than doubled between 1990 and 2016 [7].

*Lutzomyia longipalpis* responds to a variety of different chemical cues; host odour kairomones to locate blood meal sources [8], oviposition pheromones and environmental kairomones to locate oviposition sites [9–11]; and male produced sex-aggregation pheromones to locate mating sites and potential mates [3,12]. The *Lu*. *longipalpis* species complex sex-aggregation pheromones are molecules with a 16-carbon (C16) terpene skeleton (molecular weight 218 amu) or 20-carbon (C20) skeleton (molecular weight 272 amu) released by males from glandular areas which appear as large pale spots on abdominal tergites 4 or 3 and 4 [3]. Each member of the complex produces a distinctly different sex-aggregation pheromone and although the taxonomic status of the complex is not settled [13], at least 4 different members can be distinguished [14–18]. In addition, the timing of sex-aggregation pheromone release, behavioural response of conspecifics, quantities of pheromone biosynthesised and released all differ between members of the complex [19]. The most geographically widespread member of the complex produces (*S*)-9-methylgermacrene-B (C16) [3,20] and the expanding distribution of this chemotype in Southern Brazil is coincident with the emergence of VL in São Paulo State [21].

Sand fly sex-aggregation pheromones and other semiochemicals offer new opportunities for vector monitoring and control through the deployment of novel trapping and other intervention strategies [19,22,23]. A cluster-randomised control trial (RCT) of synthetic (*±*)-9-methylgermacrene-B [24] formulated in a long-lasting controlled release device [25,26] co-located with sprayed microencapsulated λ-cyhalothrin in chicken roosting sites significantly reduced *Lu*. *longipalpis* densities, and subsequently canine *Leishmania* parasite infection incidence, tissue loads and canine seroconversion incidence [22], indicating the potential of this strategy for reducing infection and VL disease incidence.

In order to optimise the impact of sex-pheromone-based control strategies on the vector population and the consequent potential impact on canine and human disease incidence, it is important to understand the role of the pheromone in *Lu*. *longipalpis* mating dynamics. Field studies have shown that the rate at which both males and females are attracted to synthetic pheromone increased asymptotically as the quantity of pheromone increased linearly and that the sex-aggregation pheromone was important in maintaining aggregations of both sexes [27]. These results suggest that pheromone and insecticide combinations would be more effective when the amount of pheromone is relatively low and if trap spacing is optimised. Therefore, to determine the spacing between the pheromone traps it is important to know the distance over which the pheromone might attract female and male *Lu*. *longipalpis*.

Mark-release-recapture studies on the dispersion of *Lu*. *longipalpis* indicate that they mostly remain in the area in which they were initially captured and it is likely that this loyalty is driven by the male sex-aggregation pheromone [28–31]. However, individuals can travel relatively long distances from the release site, e.g. 1 male and 1 female were found 175 and 243m respectively from the release point after 14 days [28] and other studies suggest that *Lu*. *longipalpis* may travel even further (i.e. up to 700m) over the course of many nights [30]. However, these studies did not specifically measure attraction to a source of pheromone and the maximum distances recorded are atypical of the population where the majority (>92%) of the marked flies were recovered at the release sites [28].

Previous laboratory studies showed that female *Lu*. *longipalpis* were attracted to sex-aggregation pheromone extracted from male sand flies and co-located with a live anaesthetised hamster, over a distance of 2.4m [32]. Field experiments in Brazil showed that a combination of synthetic sex-aggregation pheromone and host odour (chicken) attracted female and male *Lu*. *longipalpis* over at least a few metres, but these distances were limited in accordance with the experimental design [25,26,33]. However, no studies have been carried out to specifically determine the distance over which the synthetic pheromone might be attractive to *Lu*. *longipalpis* in the field. Therefore, we carried out a series of mark-release-recapture (MRR) experiments to determine the distance over which both male and female *Lu*. *longipalpis* might be attracted to a source of synthetic sex-pheromone. This study also assessed the ability of the pheromone to attract sand flies over several consecutive nights.

## Material and methods

### Study area

The study was carried out in Governador Valadares (Minas Gerais State), a city of approximately 280,000 inhabitants on the Rio Doce in Southeast Brazil. The region has a tropical wet and dry savanna/climate (Köppen-Geiger classification: Aw) with a pronounced dry season from May to September and wet season from October to April. The study area is surrounded by seasonal semideciduous forest and savannah which includes areas that are protected because of their wildlife and landscape importance.

**Fig 1:**
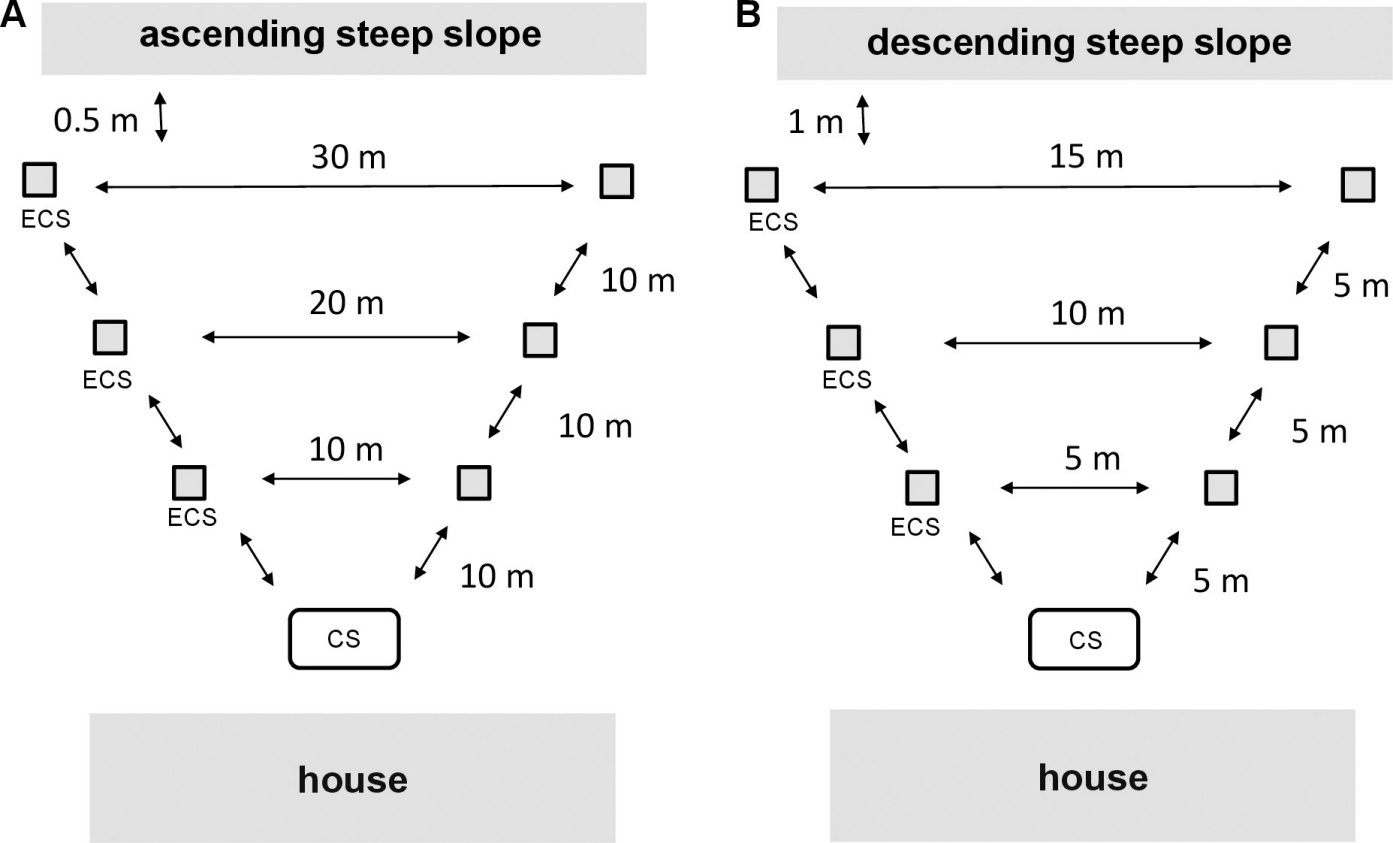
Trapping layout at household A and household B. Experimental chicken sheds (ECS) at 10, 20 and 30 m (household A) and at 5, 10 and 15 m (household B) from the chicken shed (CS) release point, respectively. The diagram also shows local topographical features; at site A there was a steep (20°) ascending slope beyond the furthermost traps. At site B there was a steep (12°) descending slope beyond the furthermost traps.

Experiments were conducted in two households: Chácara Recanto de Cachoeira (household site A), 18°53'56.4" S; 41°56'09.6" W and Village da Serra (household site B), 18°52'18.6" S; 41°55'54.9" W (Fig 1). Both sites were located on the edge of the city and had different physical and ecological characteristics (Table 1). The households were chosen because they had moderate to high densities of *Lu*. *longipalpis*, large open areas free from the presence of obstructions that might break up the pheromone plume, and that offered daily accessibility to operate.

**Table 1:** Physical characteristics of the household sites where the MRR experiments were carried out in Governador Valadares (Minas Gerais, Brazil).

Household		Chicken roost	Number of roosting chickens	*Lu*. *longipalpis* density *	Description of site
A	Chácaras Recanto de Cachoeira	Semi-open wooden shed (2.5 x 2.0m)	ca. 20	79±50	A large residence situated at the lower part of a steep incline. The household was on a rocky area with the chicken shed situated opposite the steep slope. The terrain where the experimental chicken boxes were placed was a slope gently ascending towards the steep incline. The steep slope delimited the maximum distance of study towards the north, west and east.
B	Village da Serra	Open tree branches and ladder (3.0 x 3.0m)	ca. 12	13±7	A medium sized residence was situated on a small hill. The household chicken shed was located at the top of the hill and the terrain where the experimental chicken sheds were placed descended gently for 20 m after which the slope dropped away steeply.

* Average (mean ± SEM) number of *Lu*. *longipalpis* trapped over four consecutive nights prior to experiments by CDC-traps with pheromone dispenser situated at the chicken roost.

### Fluorescent powders and marking apparatus

**Fig 2:**
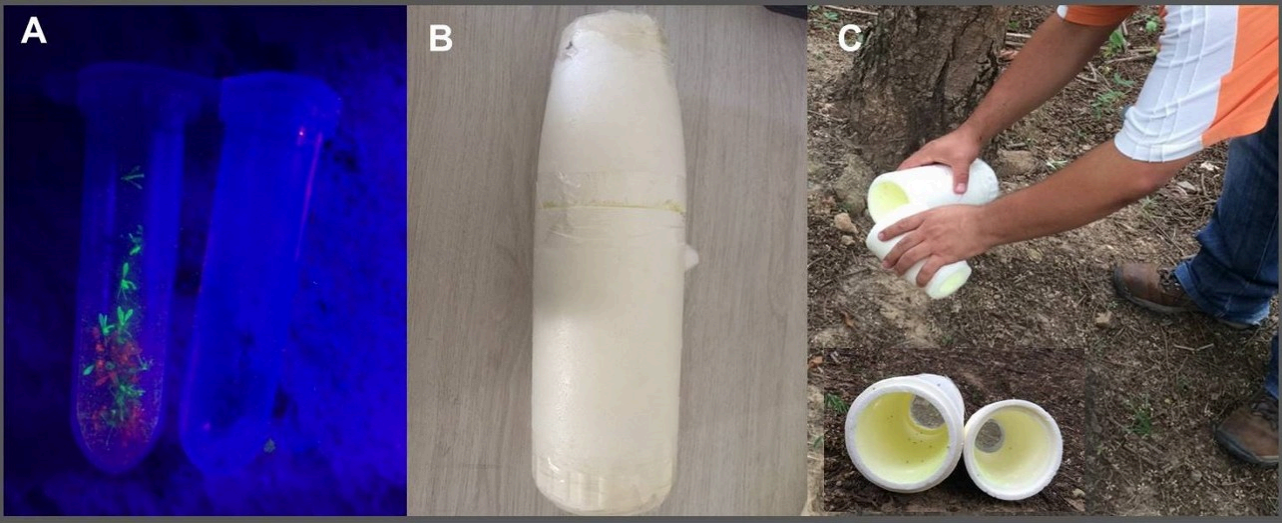
Mark-release-recapture experiments. (A) marked (lime and pink) *Lu*. *longipalpis* sand flies (left side) and unmarked ones (right side) under UV lighting, (B) home-made tapered cylinder device, and (C) release of sand flies in the field (insert lower left showing the two parts opened for quick release of the contents).

It has been reported that some fluorescent powders are toxic [28], therefore in order to stablish which powder to use in the MRR experiments we determined the effect of two different brands of powders on sand fly longevity. Four colours were trialled; lime and pink (Ultra Glow dark fluorescent powder, KilaBitzzz, Wigan, UK) and orange and white (Superior quality fluorescent luminous glow UV, Visual Bliss UV paint, Ashburton, UK). These pigments are micronized fluorescent powders that glow under UV lighting (Fig 2A).

A device to mark the sand flies with the powders was manufactured with some modifications (Fig 2B). We used a polystyrene tapered cylinder (25cm long, 7cm diam. at one end and 4cm. diam. at the other end). The wide end had a large opening (6cm i.d.) and the narrow end a smaller opening (3cm i.d.). These open ends were covered with fine nylon mesh, glued in place. The tapered cylinder was constructed from two-equal length parts that were push-fitted together in the middle and were easily separated for quick release of the sand flies. A small hole (1cm diam.) was drilled in the middle portion of one of the sections of the tapered cylinder to allow the introduction of the sand flies. After introduction of the sand flies, this hole was blocked with a small piece of cotton. Air was pumped via a rubber suction bulb attached to a plastic tube (5cm long) through the nylon mesh at the narrow end of the tapered cylinder which served as an air/fluorescent powder distribution compartment. A total of 0.2g of fluorescent powder, placed inside the rubber bulb, was uniformly blown from the narrower opening (3cm diam.) through the entire marking device (3 puffs were necessary to deplete the powder from the marking device). The large opening allowed the excess fluorescent powder that did not attach to the sand fly bodies to be removed. We used a separate tapered cylinder for each colour.

### Effect of the fluorescent powders on the sand flies

To determine the effect of possible differences in the fluorescent powders on their effectiveness to mark the sand flies and their effect on mortality, we carried out preliminary validation experiments using males and females together. These preliminary experiments were carried out on field-collected *Lu*. *longipalpis*.

To assess the effect of the powder on mortality, 75 specimens (mixed males and females) in three replicates (25 per group) were placed inside the polystyrene tapered cylinder where they were marked with fluorescent powder, either lime, pink, orange or white. After marking, the sand flies were then transferred to fine mesh nylon cages (30 x 30 x 30cm). They were supplied with sugar (25% fructose solution on small pieces of cotton wool) and water (damp gauze on the top of the cage). The cages were placed inside a semi-opaque plastic bag to maintain humidity. Temperature was kept stable thorough the laboratory trials (29 ± 2°C) and relative humidity (rh) was maintained at 80 ± 10%. Mortality rates were recorded at different time intervals post marking (1min, 2h, 4h, 12h and then at 12h time periods until 72h). A group of unmarked sand flies were held in a separate cage as a control.

To assess effectiveness and longevity of powder coverage, only the lime and pink powders were used in this test as the orange and white powders were shown in the previous test to cause significant lethality. A total of 60 sand flies (mixed males and females) in two replicates (groups of 30) were powder coated in the manner previously described, with each colour powder. They were killed by placing in a freezer (-20°C) after 2h and 72h post-treatment and stored in 70% ethanol [34] until they were examined in a dark room under LED UV illumination with a dissecting microscope to assess the coverage of powder over time. Coverage was recorded as i) none (no powder), ii) very poor (1–3 particles of fluorescent powder), iii) below average (3–10 particles), iii) average (10–30 particles), iv) above average (body partially covered), and v) excellent (body completely covered).

### Mark-release-recapture (MRR) trials

Collection and marking of *Lu*. *longipalpis* were conducted at two peridomestic households (A and B) from 12^th^ September 2016 to 15^th^ February 2017 (average mean temperature: 23.1°C and 370mm precipitation).

On the evening of day 1, either three or four downdraft traps (CDC miniature light trap model 512, John W. Hock Company, Florida, USA) fitted with a synthetic pheromone lure (no light) were set up around the household to trap male and female sand flies. The species-specific attraction of the synthetic pheromone ensured that only the target insect *Lu*. *longipalpis* was attracted [26]. The next morning (day 2), the captured sand flies in their collection cages (30 x 30 x 30cm), were returned to the laboratory and kept at 80±10% rh and given 25% fructose solution *ad libitum* until the afternoon of day 2 when the live sand flies were sexed, counted and transferred to the marking device, where they were held for no more than two hours before being released in the field. Dead or damaged specimens that were unable to fly properly were discarded from the marking device.

In the field, a pair of plywood experimental chicken sheds [26,27,33] were used to recapture the marked and released *Lu*. *longipalpis*. A chicken from the householder’s flock was placed in each experimental chicken shed and given food and water *ad libidum*. The experimental chicken sheds were separated from each other by the same distance that they were separated from the release point, i.e. an experimental shed placed 5m from the release point was 5m from the other experimental chicken shed (Fig 1). A downdraft trap (Hoover Pugedo, HP Biomédica, Minas Gerais, Brazil) [35] (without light), powered by a 6V rechargeable battery was placed in each experimental shed. One experimental shed (test) was fitted with a “lure” loaded with 10mg of the synthetic sex-aggregation pheromone [(±)-9-methylgermacrene-B; CAS 183158-38-5)] while the other shed was identical but without the pheromone (control). On the evening of day 2, the marked sand flies were released in the householder’s chicken shed. The polystyrene marking device with the marked sand flies was opened and left for a few minutes in the horizontal position for all the marked sand flies to leave (Fig 2C). Trapping in the experimental chicken sheds (test and control) was then subsequently conducted consecutively over three nights (days 2, 3, and 4). The experimental sheds were kept in the same position during this time. Each morning the sand fly collection cages containing the night’s sand fly catch were removed, the chickens were released, and the trap batteries replaced. The pheromone lure was removed during the weekend (days 5, 6, 7), and kept in the freezer at -20°C. Pheromone lures were replaced after 6 days of use. The next week (day 8), a new batch of sand flies was trapped, marked and then released (day 9) but the position of the test and control sheds was reversed and the subsequent trapping was conducted over three consecutive nights (days 9, 10 and 11). The lime and pink fluorescent powders were alternated weekly. Thus, each distance; 10, 20, and 30m was tested twice at site A and distances 5, 10, 15m were tested twice at site B. Each distance was tested for two weeks and therefore all distances for six weeks for each household (Fig 1). The distances were tested independently, i.e. traps were placed at 10m only, for 2 weeks, then traps were placed at 20m only for 2 weeks and finally at 30m only for 2 weeks. The same approach was taken for 5, 10 and 15m distances. Experiments were carried out on calm nights when there was no discernible wind. On occasions when heavy rain or strong wind disrupted a night’s collection then the whole experiment for that week was terminated and the experimental replicate for that week was repeated from the beginning. Data concerning wind (average speed, maximum speed, direction) obtained from the National Institute of Meteorology of Brazil (https://mapas.inmet.gov.br/) is provided for each site (S1 Table).

### Statistical analysis

The range of attraction is defined as the distance over which a sand fly could travel from the point of release to the point of capture in a synthetic sex-aggregation pheromone baited trap. The recapture rates were calculated from the number of marked sand flies recaptured divided by the number of marked sand flies released x 100. The proportion of male and female sand flies recaptured was compared with the released ratio. After assuming data were not-normality distributed, differences in the number of recaptured sand flies at the three different distances for both residences and between nights were compared using the non-parametric Kruskall-Wallis test (IBM SPSS statistics v. 23.0).

Fluorescent powder survival curves were presented as a Kaplan-Meier plot. Survival times of the four colours were first compared against the untreated control and then analysed in paired comparisons between untreated and treated sand flies for both fluorescent brands using the log-rank test. The level of coverage of lime and pink powder over time was compared using chi-square analysis (R software v 2.0).

### Ethics statement

The project, including the involvement of householders, was reviewed and approved by the Faculty of Health and Medicine Ethical Review Committee (FHMREC15125) at Lancaster University. This study was carried out in accordance with the guidelines of the Animals in Science Regulation Unit (ASRU) and in compliance with the Animals (Scientific Procedures) Act (ASPA) 1986 (amended 2012) regulations and was consistent with UK Animal Welfare Act 2006 and The Welfare of Farmed Animals (England) Regulations 2007 and 2010. Oral consent was obtained from the Governador Valadares health authority (CCZ) to conduct the study within their administrative jurisdiction and from both of the householders for use of their animals and property.

## Results

### Fluorescent powder trials

#### Survivorship treatment

**Fig 3:**
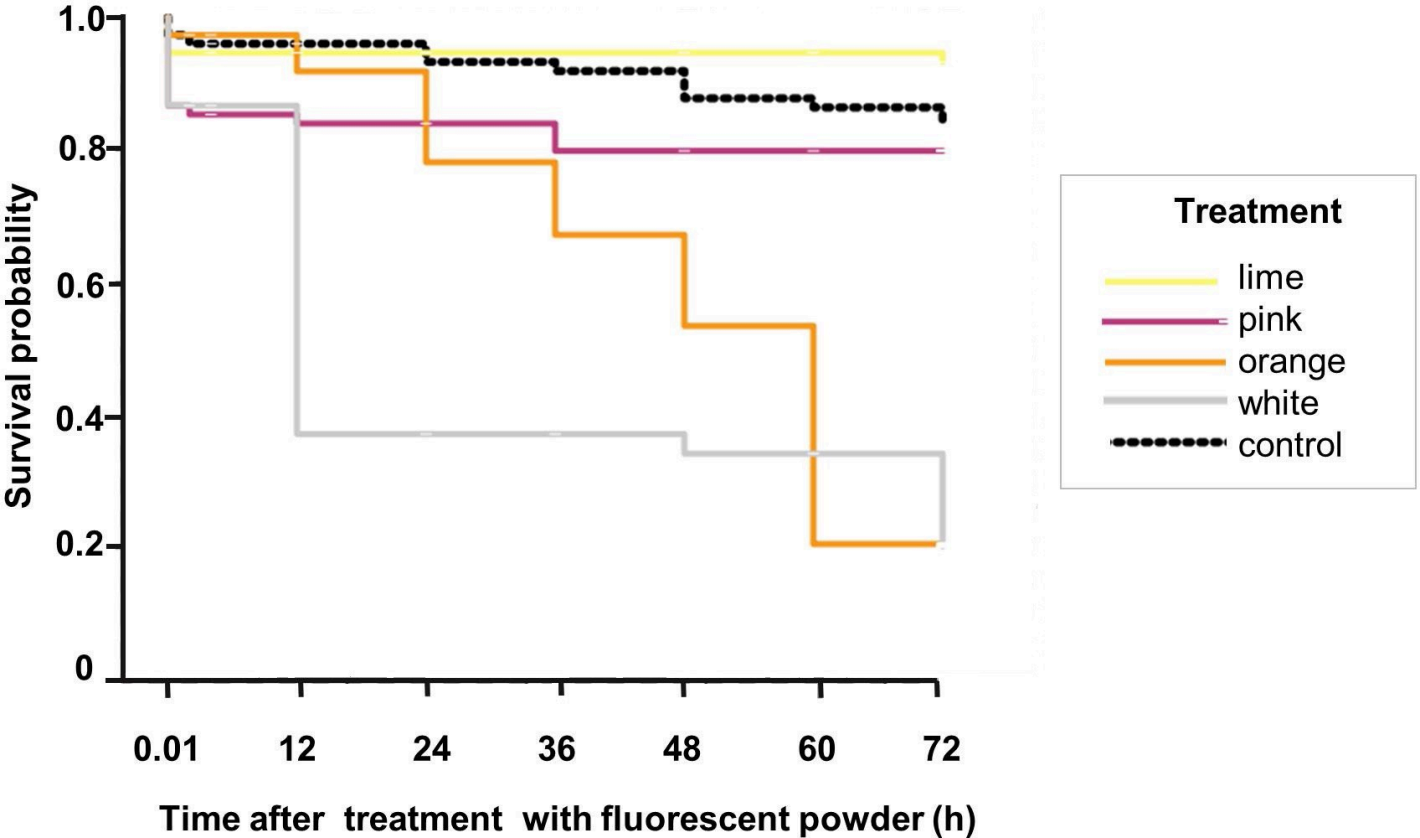
Kaplan-Meier survival curves for *Lu*. *longipalpis*. Sand flies were marked with one of four fluorescent powders (lime, pink, orange or white) and their survival over 72h in laboratory conditions was recorded. Control sand flies (black) were not marked with fluorescent powder but were similarly observed for 72h.

Overall, the fluorescent powder caused significant mortality in treated *Lu*. *longipalpis* sand flies compared to the control (Log-rank test, χ^2^: 162.9, df = 4, *P* ≤ 0.001) (Fig 3). There was no significant difference in the mortality of the untreated control *Lu*. *longipalpis* compared to those treated with either lime or pink powders (lime, χ^2^: 2.1, df = 1, *P* = 0.146 and pink, χ^2^: 0.6, df = 1, *P* = 0.449). Although a relatively small number of sand flies died over time [72h: lime = 6/75 (8%) and pink = 15/75 (20%)] a similar tendency was observed in the unmarked controls [12/75 (16%)]. Significant differences in the survival of sand flies powdered with either orange or white fluorescent powders compared to the untreated control were observed (orange, χ^2^: 52.8, df = 1, *P* ≤ 0.001 and white, χ^2^: 62.2, df = 1, *P* ≤ 0.001) (Fig 3). After 12h there was a noticeable decline in the survival rate of *Lu*. *longipalpis* treated with the white fluorescent powder and more than 75% of the sand flies had died after 72h with both white and orange powders (Fig 3), making its use unfeasible in further MMR trials.

#### Coverage treatment

**Fig 4:**
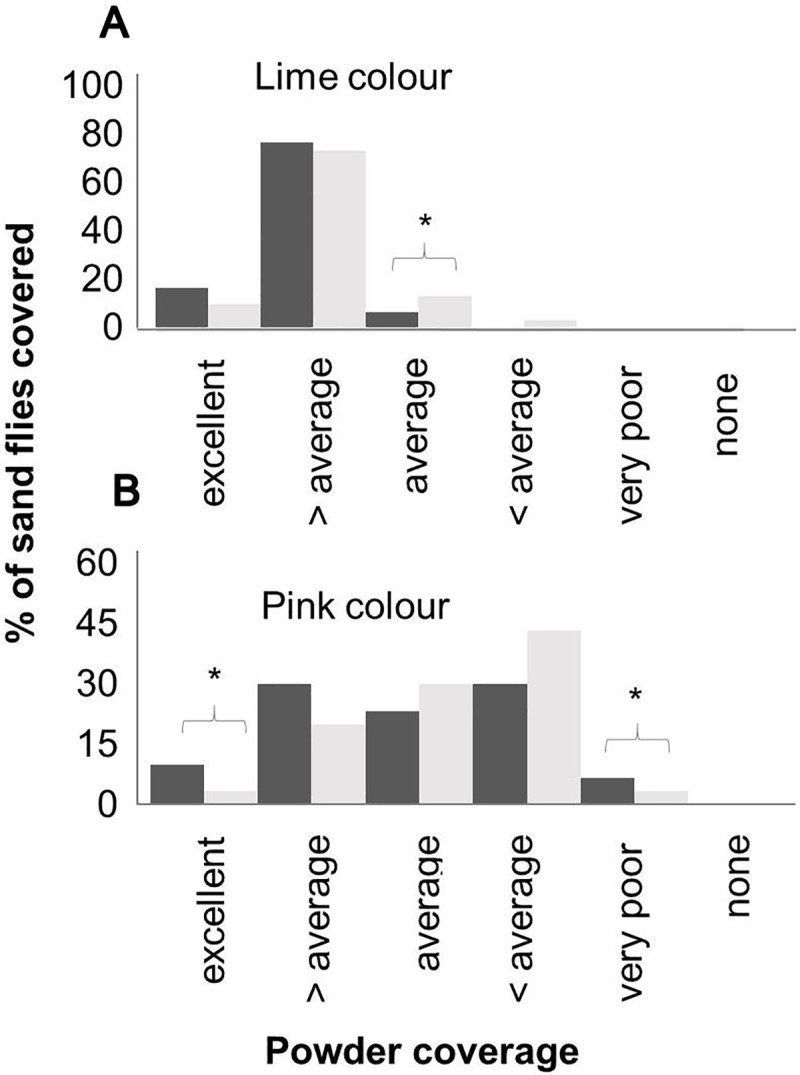
**Percentage of *Lu*. *longipalpis* sand flies covered with lime (A) and pink (B) fluorescent powders.** The dark bars represent the percentage of sand flies covered 2h post powder treatment and the light bars 72h post powder treatment. The coverage is graded by observation as i) excellent (body completely covered), ii) above average (body partially covered), iii) average (10–30 particles), iv) below average (3–10 particles), v) very poor (1–3 particles of fluorescent powder) and vi) none (no powder). * Denotes significantly different (*P* ≤ 0.05).

There was no difference in the coverage of the sand flies 2h or 72h post-treatment for either the lime or pink powders, with a few exceptions (Fig 4). Both powders adhered to all sand flies for the length of time of the experiment, showing greatest attachment to the abdomen, thoracic region and wings. The lime powder adhered better than the pink as indicated by the higher proportion of sand flies (70–75%) that were heavily marked and no specimens with a low level of marking (Fig 4).

### Mark-release-recapture trials

#### Capture of unmarked-sand flies

In total, 1834 (1392 males and 442 females) sand flies were captured at the two experimental sites (Table 2). Overall, captured male sand flies were 3x more numerous than female sand flies. In the control traps, very few *Lu*. *longipalpis* (25 at site A and 3 at site B) were captured (Table 2).

**Table 2:** Summary of numbers of *Lu*. *longipalpis* sand flies captured and recaptured at the two household sites (A and B) in Governador Valadares (Minas Gerais, Brazil).

**A**	marked and released			captured (unmarked)						recaptured (marked)					
				control			pheromone			control			pheromone		
	m	f	m+f	m	f	m+f	m	f	m+f	m	f	m+f	m	F	m+f
10m	100	35	135	12	8	20	380	147	527	0	0	0	12	2	14
20m	171	71	242	2	1	3	401	138	539	0	0	0	32	4	36
30m	283	146	429	0	2	2	482	109	591	0	0	0	13	2	15
**tot**	**554**	**252**	**806**	**14**	**11**	**25**	**1263**	**394**	**1657**	**0**	**0**	**0**	**57**	**8**	**65**
**B**	marked and released			captured (unmarked)						recaptured (marked)					
				control			pheromone			control			pheromone		
	m	f	m+f	m	f	m+f	m	f	m+f	m	f	m+f	m	F	m+f
5m	299	121	420	1	2	3	30	16	46	0	0	0	5	0	5
10m	110	92	202	0	0	0	36	7	43	0	0	0	2	1	3
15m	198	78	276	0	0	0	48	12	60	0	0	0	0	0	0
**tot**	**607**	**291**	**898**	**1**	**2**	**3**	**114**	**35**	**149**	**0**	**0**	**0**	**7**	**1**	**8**

Household A and B; m: males. f: females. Marked and released: Number of *Lu*. *longipalpis* marked with fluorescent powder in the laboratory and then released in the householder chicken shed. Captured (unmarked): Number of *Lu*. *longipalpis* captured in the pheromone baited and control chicken sheds at 10, 20, 30m and 5, 10, 15m. Recaptured (marked): Number of *Lu*. *longipalpis* marked with fluorescent powder, and thus recaptured in the pheromone baited and control chicken sheds at 10, 20, 30m and 5, 10, 15m. Control = experimental chicken shed with a chicken only. Pheromone = experimental chicken shed with a chicken plus a pheromone lure.

#### Recapture of released marked-sand flies

In total, 1704 (1161 males and 543 females) were marked and released at the two experimental sites (Table 2). Subsequently, 1907 *Lu*. *longipalpis* were captured at both sites and only 73 (4.3%) were marked. At site A, 65 *Lu*. *longipalpis* (57 males and 8 females) were recaptured; 14 (10.4%) at 10 m, 36 (14.8%) at 20 m, and 15 (3.4%) at 30 m (Table 2). At site B, only 8 *Lu*. *longipalpis* were recaptured (7 males and 1 female); 5 (1.1%) at 5 m and 3 (1.4%) at 10 m. None were recaptured at 15 m (Table 2). Although twice as many males as females (2.1♂:1♀) were released, overall marked male sand flies were 7x more numerous in pheromone-baited traps than marked females at both sites. At site A, at 10m, 3x more males were recaptured than females, at 20m, 4x more males were recaptured, and at 30m, 3x more males were recaptured compared to the released ratio (Table 2). At site B, at 5m, 2.5x more males were recaptured than females and at 10 m, 2x more males were recaptured as released (Table 2). No marked sand flies were recaptured in the control traps (Table 2).

#### Recapture of marked-sand flies over distance

**Fig 5:**
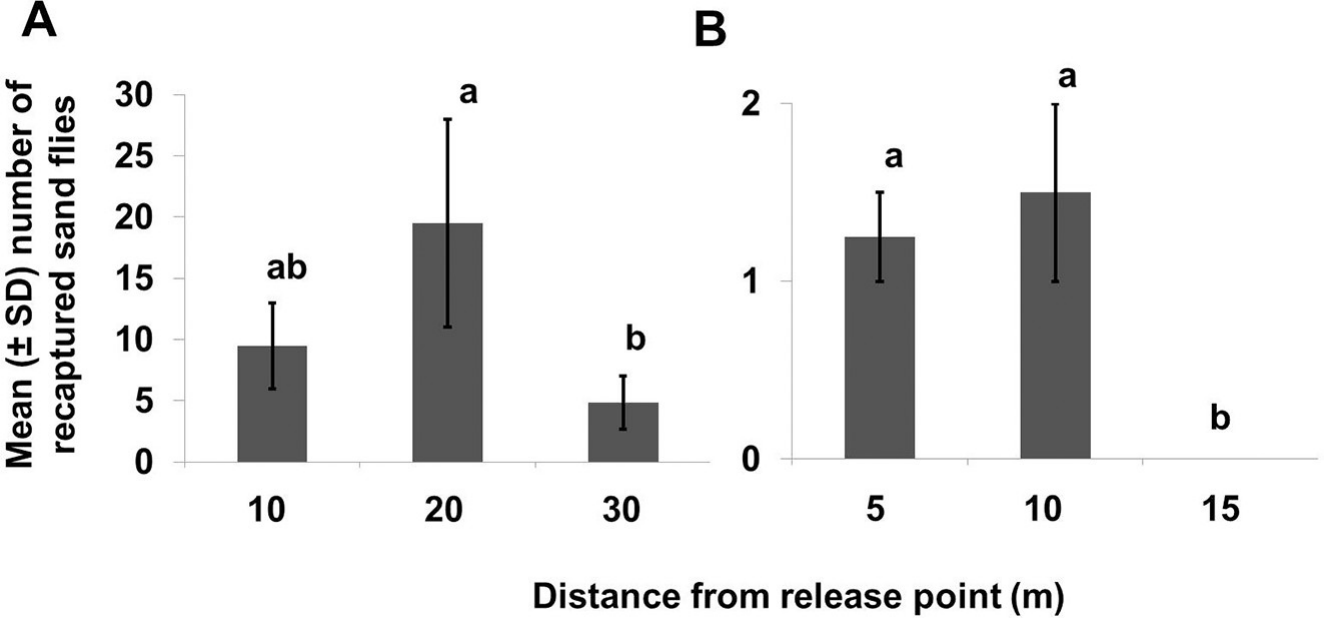
**Number of recaptured *Lu*. *longipalpis* sand flies trapped at different distances from the release point in household (A) and household (B).** The mean ± SD sand flies (males + females) for each distance from the release point was calculated from a total of two replicates (corresponding to the experiments marked with lime and pink fluorescent powders). The mean numbers of recaptured sand flies shown in bars with the same letters were not significantly different from each other (*P* < 0.05).

More sand flies were recaptured in traps placed short to medium distances from the release points at both households. At household A, the numbers of *Lu*. *longipalpis* trapped at 20m were significantly greater than the number collected at 30m from the release point (χ^2^ = 14.121, df = 2, *P* ≤ 0.05). At household B, significantly more sand flies were collected at both 5 and 10m from the release point than at 15m (χ^2^ = 16.210, df = 2, *P* ≤ 0.05) (Fig 5).

#### Recapture of marked-sand flies over time

**Fig 6:**
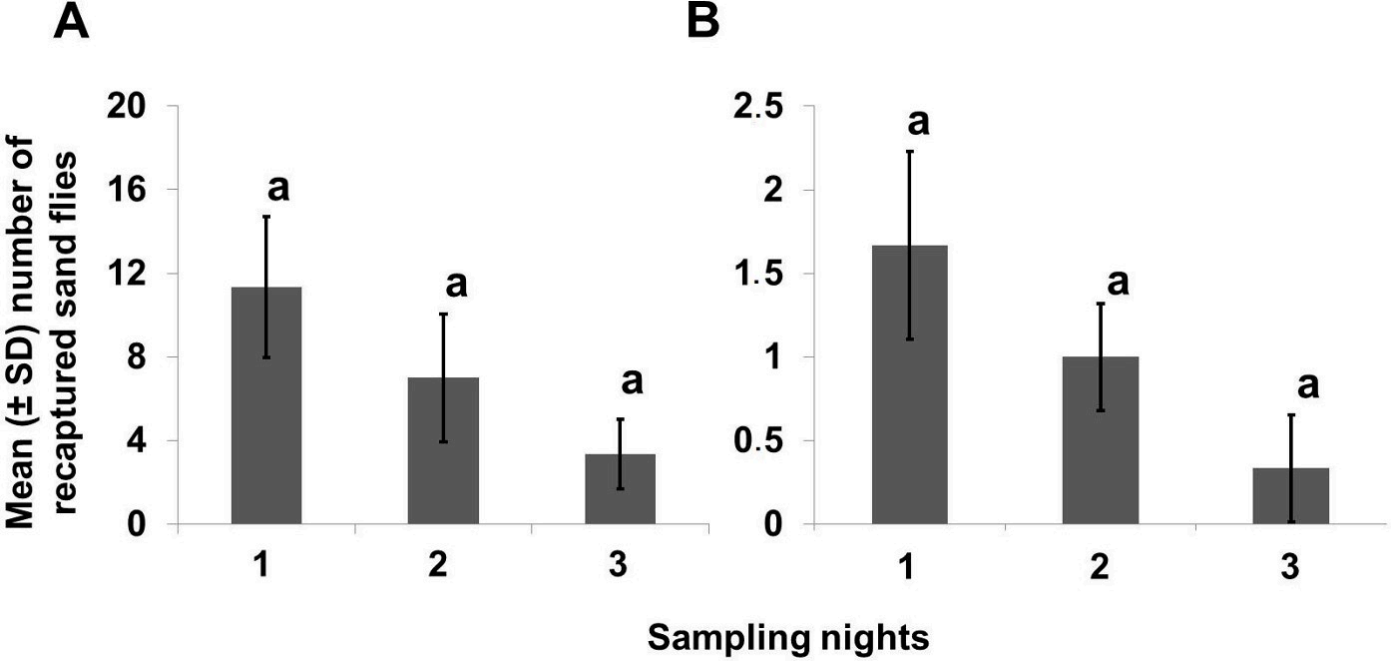
**Number of recaptured *Lu*. *longipalpis* sand flies trapped over each of three consecutive nights in household (A) and household (B).** The mean ± SD sand flies (males + females) for each night was calculated from a total of three replicates (corresponding to the experiments marked with lime + pink fluorescent powders at the three distances). The mean numbers of recaptured sand flies in bars with the same letters were not significantly different from each other (*P* < 0.05).

Overall, a higher proportion of *Lu*. *longipalpis* was collected during the first night of recapture (52% of the total), followed by the second night (32%), and (16%) on the third night. However, there were no statistical differences in capture rates between nights in household A (χ^2^ = 3.954, df = 2, *P* = 0.138) or household B (χ^2^ = 2.440, df = 2, *P* = 0.295) (Fig 6).

## Discussion

This study was the first attempt to determine the distance over which male and female *Lu*. *longipalpis* sand flies are attracted to synthetic sex-aggregation pheromone and host odour in the field. Our experiments indicated that the synthetic sex-aggregation pheromone (±)-9-methyl-germacrene-B) was able to attract individuals of both sexes from an established aggregation site to a trap 30m distant. The distance over which the sand flies were attracted varied between the two study sites. This may be because of differences in ecological, environmental, and meteorological factors [36,37] affecting pheromone dispersal, however, the numbers recaptured at site B are too low to draw firm conclusions. In this study it was not possible to find households with a chicken roosting site surrounded in all directions by equal distances of open space. Generally other barriers (buildings, other structures, vegetation) were present which added more complexity to the area around the chicken shed. Therefore, the experiments were carried out in typical chicken sheds at household sites and thus are a better approximation of what may really occur than at a location which allows an experimentally perfect but atypical design.

Only two previous studies, conducted in the laboratory, have investigated the distance over which the pheromone is attractive. The first showed that female *Lu*. *longipalpis* were attracted to pheromone presented along with hamster odour over a distance of 2.4m [32] but the response of male *Lu*. *longipalpis* was not determined. In the second experiment, males and females were shown to be attracted to synthetic pheromone over a distance of 1.7m [38]. In both experiments, the distance of attraction was limited by the size of the wind-tunnel and neither experiment was conducted on the same pheromone producing member of the complex as is found in Governador Valadares.

Our study showed that *Lu*. *longipalpis* sand flies were attracted from distances between 5 to 30m in a few hours (over a single night) by the synthetic sex-aggregation pheromone lure (plus host-odour). This pattern of long-distance attraction to the synthetic sex-pheromone lure had also been observed previously in incomplete preliminary trials in Campo Grande (Mato Grosso do Sul) and rural environments of Governador Valadares. For many large moths, sex pheromone attraction can be over tens of metres or even kilometres under favourable conditions [39–42]. Dipteran pheromones typically attract over short range (3-5m) and long-distance attractant sex pheromones are not common, but where they occur, they are female produced and attract males only [43]. The sex pheromone of the female apple leaf midge, *Dasineura mali* (Diptera: Cecidomyiidae) for example, produces a sex pheromone that is attractive over at least 50m [44]. Male tephritids (Diptera: Tephritidae) also produce a long-distance sex pheromone, e.g. *Bactrocera cucurbitae* is estimated to attract conspecifics over 27m dependant on environmental and other conditions [45].

In this study, the pheromone in the experimental chicken shed attracted both female and male *Lu*. *longipalpis* from the natural chicken shed. This would be a significant benefit in reducing the peridomestic *Lu*. *longipalpis* population but appears to contradict previous findings that showed that traps containing five pheromone lures did not attract sand flies from traps containing one pheromone lure even when placed 5m apart [27]. This could be explained by differences in the pheromone release dynamics of the synthetic pheromone lure which releases pheromone at continuous, relatively stable state, and the natural male aggregation which may regulate pheromone release depending on as yet undetermined factors; time, density, etc.

The decline of recapture rates as distance from the release point increases is frequently reported in other studies, e.g. moths [36,46], fruit flies [47], and beetles [48,49], although the findings depend on dispersal activity of the subject insect after release [50]. The relationship between distance and recapture rates is frequently not linear with more recaptures close to the release point. This can partially be explained as a result of the decreasing density of the target insect in a larger volume as distances from the release point increases (the volume of the hemispherical space around the release point is 27x greater when the trap is 30m from the release point compared to 10m) and a tendency for the insects to settle nearer the release point [28,50]. Unfortunately, we did not sample inside release chicken sheds to verify sand fly site-loyalty. In this study as in others [30], sand flies were predominantly captured during the first night after release indicating that the synthetic pheromone was able to attract sand flies in a relatively short time period. Although the rate of trapping unmarked sand flies was maintained during the three trapping nights there was a noticeable decrease (although no statistical difference) in recapture rates of marked sand flies over the next two consecutive nights. This drop was likely to be the result of the natural dispersion of *Lu*. *longipalpis* [28] as no decrease would be expected to occur because of a loss of the effectiveness of the synthetic pheromone lures, as they are attractive for up to three months [26]. The large differences in the number of wild unmarked male and female flies captured at the pheromone treated compared to untreated recapture chicken sheds demonstrates the general attractiveness of the synthetic pheromone. Host odour attracts female sand flies and is a synergist for the sex- aggregation pheromone [51], and thus a chicken was placed in both of the experimental (pheromone present) and control (no pheromone) sheds. No marked sand flies were collected in the control sheds and they were collected only in pheromone-baited sheds. Thus, the host odour by itself provided an indication of the natural dispersion of the sand flies (unmarked specimens) and it was therefore interesting to note that there was little difference in the numbers of males or females trapped at 10, 20, or 30m from the release point at site A, or 5, 10 or 15m from the release point at site B. The household sites A and B were carefully chosen to exclude any potential focal points for sand fly aggregations other than the release points themselves and suggest that *Lu*. *longipalpis* are more homogeneously distributed in the environment than has previously been recognised [27].

In both CDC light-trap and pheromone-trap collections in real chicken sheds the ratio of males to females has typically been 4♂:1♀ [25,26]. In this study, considering that sand flies were released in the ratio, ca. 2♂:1♀, a similar ratio of males to females might be expected in the pheromone-baited traps. However, marked flies were recaptured in the ratio of approximately 7♂:1♀ at both sites. Considering that blood-seeking female *Lu*. *longipalpis* sand flies are more mobile than males (that do not blood-feed) [29], we might expect to recapture more females than males in downdraft-baited traps. However, the numbers suggested a gradual decrease in the proportion of marked females caught as the distance from the release point increased. The reasons for this change are unclear but may be related to a change in the physiological state of females after they have obtained a blood-meal in the natural chicken shed. The ratio of unmarked males to unmarked females did not change as much over distance. It is therefore possible that *Lu*. *longipalpis* males are more responsive than females to the smaller amount of pheromone released from traps that are further away and that the traps are also capturing unmarked sand flies from their immediate vicinity. This distinctly intraspecific difference in attraction may also be related to different types of olfactory receptors for anemotaxis between sexes [52].

Recapture rates from the site A differed notably from site B. Clearly there are important factors that can have a substantial impact on the sand fly population and their ability to respond to the synthetic pheromone. There are many different possible explanations for the marked differences observed in the distance that the males and females would travel and the recapture rates at the two sites. Attraction and capture of an insect is a complex process involving multiple components, several critical elements may individually or collectively be important; i) spatial memory/olfactory memory: one possibility is that sand flies were capable of learning a familiar area map to facilitate movement and specific routes between feeding, resting and breeding sites [53–56], ii) population density: a natural aggregation in the chicken shed producing large quantities of pheromone would compete with the synthetic pheromone, causing a reduction in the number of specimens attracted to pheromone-baited traps [53,57], iii) fidelity to specific hosts might influence the recruitment of sand flies in locations in which there is an availability of sources of blood [53,55,58], and iv) abiotic factors could explain the dispersal of *Lu*. *longipalpis* at the two sites, in particular high wind speed has been attributed to decreasing or failing collections as it interferes with sand fly flight [59–61]. Wind strength and prevailing wind direction would affect the gradient, diffusion, and aerial distribution of the pheromone plume which in turn could influence the sand flies ability to locate the pheromone source [62,63]. Dispersion of pheromone would also be influenced by temperature, humidity, topography, construction of the pheromone release device (lure) and formulation of the pheromone within the release device as well as the design, construction and placement of the trap [64, 65]. According to meteorological data, site B was exposed to more wind gusts than site A, however this only occurred for short periods of time. The predominant wind conditions were gentle at both households during the night. There are also environmental variables such as habitat (e.g. presence of vegetation and alternative host animals) which potentially play a critical role in shaping the response to pheromones [28,37,60,66].

The fluorescent powders used in this study provided a quickly identifiable marker for tracking the movement of both male and female *Lu*. *longipalpis*; they are inexpensive, readily available, environmentally safe, and easily applied and detected [67]. However, our study also showed that some fluorescent powders shortened the life of the sand flies and highlighted the need to test their effect before use [30,68]. Fluorescent powders may also decrease mobility and interfere with sensory organs resulting in adverse behavioural effects [69]. In this study we showed that some fluorescent powders have a significant impact on sand fly survival, it is also possible that the powder that we selected to use may have had other more subtle effects on the sand fly behaviour and this may have altered the response to the pheromone.

This is the first attempt to measure the attractiveness of the *Lu*. *longipalpis* synthetic sex-aggregation pheromone in the field. Although male produced sex-aggregation pheromones are not uncommon in other insect groups [70], *Lu*. *longipalpis* sand flies represent an exception amongst the Nematocera where female produced pheromones are typical. This paper provides the evidence that both sexes are attracted from long distance and suggests that synthetic pheromone lures co-located with insecticide could be spaced 60m apart, which represents approximately a single trap per household in typical VL endemic communities in Brazil, with the objective to manipulate female vector blood-feeding and probability of transmission of *Le*. *infantum*. In a recent community trial to study the effect of the pheromone co-located with insecticide on the population of *Lu*. *longipalpis* in pheromone+insecticide treated houses and untreated (placebo) houses we showed that the numbers of both female and male *Lu*. *longipalpis* in the placebo traps placed on average 16m (2.2–45.2m) from pheromone-treated houses were significantly reduced by 44% and 50% respectively indicating that the pheromone is able to attract females and males over long distances under real field conditions. The repellent effect of insecticide on this distance was not determined in this study although from previous work [25] we know that the attractancy of the sex-aggregation pheromone overcomes the repellence of the insecticide.

## Conclusions

The results presented here are important for the development of strategies for monitoring and controlling *Lu*. *longipalpis* sand flies using synthetic male sex-aggregation pheromone. We determined for the first time the potential distance of attraction (up to 30m) to the sex-aggregation pheromone in combination with a host-odour for male and female *Lu*. *longipalpis*. The effectiveness of synthetic pheromone is affected by a variety of potential factors which can have a significant impact on the performance of the pheromone-baited traps. In this respect, more detailed experimental work is required, particularly on measuring factors such as micro-meteorological and environmental parameters. Further studies including mathematical models will enable more precise algorithms for determining trap placements and density of baited-traps. The development of geographic information systems and risk maps to deploy pheromone dispensers will have also significant importance for controlling this species in residences.

## Supporting information

S1 Table Wind parameters recorded from a meteorological station located in Governador Valadares (Minas Gerais, Brazil). (DOCX)
