# Supplementary material for: Synthetic sex-aggregation pheromone of Lutzomyia longipalpis, the South American sand fly vector of Leishmania infantum, attracts males and females over long-distance
Source: PLoS Negl Trop Dis. 2020 Oct 20;14(10):e0008798. doi: 10.1371/journal.pntd.0008798 (PMC7598924; doi:10.1371/journal.pntd.0008798)
Supplement: S1 Table — (DOCX) [file pntd.0008798.s001.docx]

**S1 Table.**  **Wind parameters recorded from a meteorological station located in Governador Valadares (Minas Gerais, Brazil).**

| Household | Distance (m) | Powder (colour) | Wind parameters ^1^ | | |
| --- | --- | --- | --- | --- | --- |
|  |  |  | Speed ^2^ | Max ^3^ | Direction ^4^ |
|  |  |  |  |  |  |
| A | 10 | Lime | 2.0 | 10.1 | SE |
|  |  | Pink | 2.2 | 11.8 | S |
|  | 20 | Lime | 2.2 | 8.8 | SE |
|  |  | Pink | 2.6 | 8.7 | E |
|  | 30 | Lime | 2.4 | 12.2 | SE |
|  |  | Pink | 1.7 | 11.9 | E |
| B | 5 | Lime | 2.5 | 16.1 | E |
|  |  | Pink | 2.0 | 13.9 | SE |
|  | 10 | Lime | 1.6 | 7.8 | SE |
|  |  | Pink | 1.8 | 8.0 | SE |
|  | 15 | Lime | 1.6 | 12.1 | SE |
|  |  | Pink | 2.1 | 10.6 | E |

^1^ Data in each line includes the three trapping nights for each different powder colour replicate. ^2^ Speed = average (ms^-1^) per hour; ^3^ Max = Maximum wind speed (ms^-1^) per hour recorded; 4 Direction = Predominant direction of the wind (cardinal points).
